# Circulating microRNA profile unveils mechanisms of action of acitretin for psoriasis vulgaris

**DOI:** 10.1080/21655979.2021.1925205

**Published:** 2021-05-11

**Authors:** Lin Chen, Jie Li, Ying Yao, Shanlong Wang, Shuangjin Zheng, Xinggang Ju, Bin Zhang

**Affiliations:** Department of Dermatology, The Second Affiliated Hospital of Henan University of Science and Technology, Luoyang, Henan Province, China

**Keywords:** Psoriasis vulgaris, acitretin, miRNA, MAPK, JAK-STAT, NF-ΚB

## Abstract

Psoriasis vulgaris is a common chronic and recurrent inflammatory skin disease. In clinical practice, acitretin is the first-line treatment drug for psoriasis vulgaris. MicroRNAs (miRNAs) play a vital role in the initiation and development of psoriasis vulgaris. However few studies focused on the mechanisms of acitretin in the treatment of psoriasis vulgaris from the perspective of miRNAs. Here, the expression profiles of circulating miRNAs in the plasma of 12 patients with psoriasis vulgaris before and after acitretin treatment were sequenced. Three miRNAs (miR-146a-5p, miR-122-5p and miR-21-5p) were identified using expression pattern analysis, and the levels were significantly decreased after acitretin treatment (*P*< 0.001). Receiver operating characteristic (ROC) analyses indicated that the three miRNAs have the potential to be utilized as molecular markers to evaluate the therapeutic effect of acitretin, and the values of the area under the curve (AUC) were 0.825, 0.831, and 0.796, respectively. In addition, we predicted target genes of the three miRNAs and performed signaling pathway enrichment analyses. The results demonstrated that the target genes were mainly involved in the MAPK, JAK-STAT, and NF-κB signaling pathways, which were further validated through *in vitro* experiments. In conclusion, acitretin can suppress miRNA-mediated MAPK, JAK-STAT, and NF-κB signaling pathways by decreasing miRNAs expression, thereby inhibiting the proliferation and inflammatory response of keratinocytes.

## GRAPHICAL ABSTRACT

**Figure:**
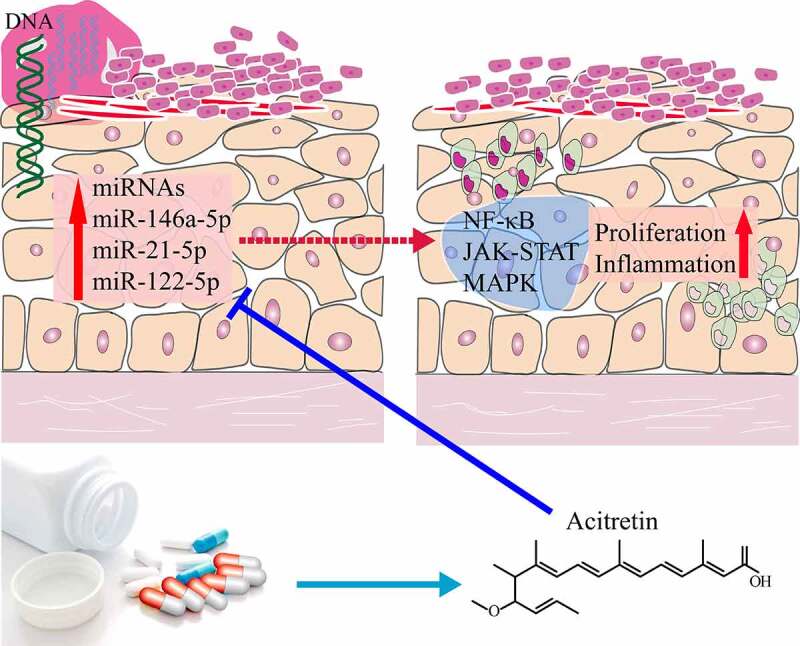

## 1. Introduction

Psoriasis, commonly known as serpedo, is a common chronic inflammatory disease of the skin. Psoriasis is caused by an autoinflammatory response induced by abnormal interactions between epidermal keratinocytes and immune cells [1]. A large number of clinical studies have demonstrated that although psoriasis is not fatal, its complications include a variety of metabolic and cardiovascular diseases, which can endanger the lives of patients [2–4]. The global incidence of psoriasis is approximately 3–4%, among which 90% of patients have psoriasis vulgaris [5,6]. Psoriasis vulgaris is a chronic and recurrent inflammatory skin disease. While the etiology is unknown, it may be related to genetic, infection, and immune dysfunction [7]. Clinically, psoriasis vulgaris is mainly treated using some anti- infectious and anti-inflammatory drugs [8,9]. Retinoid, a derivative of vitamin A, has been utilized as a treatment drug for psoriasis vulgaris since the 1970s [6,10]. Acitretin is a second generation retinoic acid drug. Due to beneficial therapeutic effects, as well as quick effect in clinical practice, acitretin has now become the first-line drug for treatment of psoriasis vulgaris in China [11]. It has been reported that acitretin plays a therapeutic role by inhibiting several signaling pathways, including JAK-STAT, NF-κB and MAPK, thereby inhibiting keratinocyte proliferation [10,11]. However, the action mechanisms of acitretin in the treatment of psoriasis vulgaris remain largely unclear.

Numerous studies have demonstrated that microRNAs (miRNAs) play a vital role in the pathogenesis of skin cancer [12] and chronic inflammatory skin diseases (i.e. psoriasis vulgaris). Many studies have shown that lesion-specific miRNA expression profile is different from that of healthy skin [13–15]. MiRNAs are a class of non-coding RNA molecules comprised of 19–25 nucleotides and can regulate gene expression at the post-transcriptional level, thus inhibiting mRNA translation or promoting its degradation [16,17]. MiRNAs are able to simultaneously regulate multiple signaling pathways and biological processes by targeting different mRNAs [18]. Studies of genes involved in the miRNA biogenesis pathways have provided evidence of the central role of miRNA in the regulation of immune response [19,20], and the dysregulation of this process can cause abnormal expression of different cytokines, which leads to inflammation and loss of immune tolerance to autoantigens, a characteristic that is common in chronic inflammatory diseases [21–23]. As miRNAs can be identified in body fluids (i.e. serum or plasma), they have become potentially useful biomarkers for risk assessment, diagnosis, and prognosis of different diseases [24]. At present, many studies have demonstrated that circulating miRNAs can be utilized as biomarkers to accurately evaluate the prognosis or diagnosis of autoimmune diseases [23,25], however few studies have investigated the mechanisms of acitretin in the treatment of psoriasis vulgaris from the level of miRNAs.

In this study, we sequenced expression profiles of circulating miRNAs in the plasma of 12 patients with psoriasis vulgaris before and after acitretin treatment. Additionally, we analyzed abnormally expressed miRNA-mediated signaling pathways, and validated these signaling pathways through *in vitro* experiments. This study revealed that acitretin can exert therapeutic function in the treatment of psoriasis vulgaris by inhibiting miRNA-mediated signaling pathways.

## 2. Materials and methods

### 2.1. Patients and sample collection

Patients with psoriasis vulgaris enrolled in this study were all from the outpatient department of Dermatology, the Second Affiliated Hospital of Henan University of Science and Technology. This study obtained ethical review permission from the hospital, and all patients and healthy subjects were asked to sign the informed consent prior to sampling. Additionally, all subjects did not have any other immune skin diseases or systemic diseases, and did not receive any immunosuppressive therapy or local treatment prior to the definite diagnosis of psoriasis vulgaris [26]. Fasting early morning peripheral blood was collected from patients who were able to meet this criteria prior to the treatment. The patients were treated with oral acitretin capsules (Chongqing Huabang Pharmaceutical Co., Ltd) once a day, for 25 mg each time for 8 weeks. After 8 weeks, fasting early morning peripheral blood was collected again. The healthy control group included subjects that underwent routine physical examination and had no previous history of psoriasis vulgaris or other immune related diseases. The healthy control group matched the age, gender and body mass index of the patient group. The detailed information of subjects that underwent miRNA sequencing is listed in Table 1.

**Table 1.** Clinical information from subjects chosen for miRNA sequencing

Parameters	Con Group	Pso Group	
	(N = 6)	(N = 12)	
Age (years)	37.25 ± 13.67	40.53 ± 11.33*^ns^*^[1]^	
Male, n (%)	3 (50%)	6 (50%)*^ns^*^[1]^	
BMI (kg/m^2^)	25.38 ± 3.76	23.73 ± 4.38*^ns^*^[1]^	
Disease duration (years)		10.68 ± 5.27	
Medication time (weeks)		8	
PASI		20.36 ± 2.03 (before)	3.87 ± 1.67 (after)***^[2]^

[1] statistically significant between Pso and Con group, *ns*: not significant

[2] statistically significant between Pso-A (8 weeks after acitretin treatment) and Pso group, *** p < 0.001.

Fresh peripheral blood obtained from the subjects was immediately injected into BD vacutainer containing a citrate anticoagulant. Next, the plasma was collected by centrifugation at 1700 g for 30 min at 4°C. The collected plasma was stored at −80°C for future use.

In addition, plasma samples of 80 healthy subjects, 70 untreated psoriasis vulgaris patients and 62 patients with psoriasis vulgaris treated with acitretin for more than eight weeks were collected for validation of expression of subsequent screening miRNAs. Detailed information of subjects is shown in Table S1.

### 2.2. RNA extraction

The miRNAs were extracted from 600 μL plasma samples utilizing miRNeasy Serum/Plasma Kit (Qiagen, Germany). The concentration and purity of miRNAs were determined by Nanodrop-1000 (Thermo Fisher Scientific, USA). The RNA purity was evaluated by the ratio of absorbance at 260 nm and 280 nm. The ratio was within the range of 1.7 to 2.0, and the concentration of RNA was greater than 100 ng/μL. The extracted miRNAs were utilized for miRNA sequencing and quantitative real-time reverse transcription polymerase chain reaction (qRT-PCR).

### 2.3. MiRNA sequencing and data analyses

The small RNA library was constructed as per the instructions of the Illumina TruSeq small-RNA sample preparation kit (Illumina, USA). The constructed library was sequenced utilizing the Illumina HiSeq 2000 platform with a single-ended 50bp (SE50) strategy. First, the LCScience ACGT10-miR v4.2 pipeline was used to remove RNA sequences of low-quality and non-miRNA sequences (mRNA, RFam, Repbase, piRNA). The reserved high-quality sequences were mapped to the human miRNA precursor sequences in the miRBase (Release 22) using Bowtie (version 1.2.2) [27]. The aligned sequences were then compared to the sequence of the human genome (Gecode, Release 36). The expression of miRNAs was normalized by RPM (Reads per million mapped reads, RPM = number of reads mapped to a miRNA ×10^6^/total number of mapped reads), and the expression of miRNAs after normalization was analyzed using the variance calculation method of DESeq2 [28]. MiRNAs with *P*< 0.05 and a two-fold difference were considered to be significant different miRNAs.

The prediction of miRNA target genes was conducted using the miRTarBase (Release 8.0) [29]. The database was designed to gather evidence-supported miRNA-target interactions, and to identify miRNA-target interactions using strong experimental evidence.

The signaling pathways of the target gene Kyoto Encyclopedia of Genes and Genomes (KEGG) were assessed utilizing the Database for Annotation, Visualization and Integrated Discovery (DAVID; https://david.ncifcrf.gov/; v6.8) [30]. Pathways with false positive rate (FDR) <0.05 and the number of genes in the pathway greater than 10 were identified using signal pathways enriched via miRNA target genes of KEGG analysis.

### 2.4. Verification of the miRNA expression by qRT-PCR

To further validate miRNA expression, the reverse transcription of miRNAs was conducted utilizing the miScript II RT kit (QIAGEN). SYBR® Premix Ex TaqTM II kit (TaKaRa, Japan) was used for quantitative PCR on the ABI 7500 instrument (Applied Biosystems, USA). The specific reaction steps were as follows: 95°C for 15 min, and then 40 cycles of reaction, which was composed of 94°C for 15 s, and 55°C for 30 s. After 40 cycles, 72°C for 30 s for extension. U6 small nuclear RNA was utilized as an internal reference for quantitative PCR. The relative expression of miRNAs was expressed as 2^−ΔΔCt^. The sequences of miRNA and internal reference primers are shown in Table S2.

### 2.5. In vitro experiment of acitretin

HaCaT is an immortalized human epidermal keratinocyte line that is often used as a cell model to study psoriasis *in vitro*. In this study, HaCaT cells were purchased from the China Center for Type Culture Collection (CCTCC, Wuhan, China) and cultured in Dulbecco’s modified Eagle’s medium (DMEM, Gibco, USA) with 10% fetal bovine serum (FBS, Gibco, USA). The concentrations of penicillin and streptomycin in the medium were 100 U/mL and 100 μg/mL, respectively. The cells were cultured at 37°C in an incubator with 5% CO_2_.

HaCaT cell suspension was seeded into a 12 well plate at a density of 2 × 10^5^ cells per well. The cells were treated either with or without acitretin (5 mol/L), respectively. After 24 h of treatment, the cells were collected for subsequent western blot (WB) experiments.

### 2.6. Western blot

These cells were lysed using RIPA buffer (Solarbio, China) for 30 min on ice, and then centrifuged at 4°C at 1 2000 rpm for 10 min to collect total protein. The extracted protein was quantified through the Pierce™ BCA Protein Assay Kit (ThermoScientific, USA). The protein was separated using a 12% SDS-polyacrylamide gels electrophoresis (SDS-PAGE), and transferred onto a polyvinylidene difluoride (PVDF) membrane (Millipore, USA). The PVDF membrane containing protein was blocked using 5% skim milk at a room temperature for 2 h. Next, STAT3, pSTAT3 Y705, NF-κB p65, ERK, pERK, and GAPDH (antibodies were purchased from Abcam, UK) specific primary antibodies were added and incubated overnight at 4°C. After incubating with primary antibodies, the PVDF membrane was washed using Tris-buffered saline-Tween (TBST, Solarbio, China), and incubated at room temperature with horseradish peroxidase (HRP)-conjugated secondary antibody (goat anti-rabbit or anti-mouse IgG, Santa Cruz, USA) for 1 h. The immunoreactive bands were visualized using enhanced chemiluminescence kit (Millipore, USA), and protein bands were quantitatively analyzed through the Image J software (NIH, USA).

### 2.7. Statistical analyses

SPSS 19.0 (SPSS Inc., USA) was used for statistical analyses of data, and the data were expressed as mean ± standard deviation (M ± SD). The Student’s t-tests and one-way analysis of variance (ANOVA) were used for comparison between two groups and multiple groups, respectively. The Binary Logistic regression model in SPSS was used to analyze the receiver operating characteristic (ROC) curve and calculate the area under the ROC curve (AUC) in order to determine the sensitivity and specificity of candidate miRNAs as markers for diagnosis of psoriasis vulgaris and acitretin treatment. R (version 3.6.0) was used to draw the curve. *P*< 0.05 was considered to be significantly different.

## 3. Results

### 3.1. MiRNA sequencing and identification

In order to investigate the change characteristics of miRNAs in the plasma of patients with psoriasis vulgaris prior to and after acitretin treatment, we collected plasma samples from 6 healthy subjects and 12 patients with psoriasis vulgaris before and after acitretin treatment. The information for each subject is shown in Table 1. The age, gender, and body mass index of patients with psoriasis vulgaris are matched to those of healthy subjects. The The psoriasis area severity index (PASI) of patients was significantly decreased after treatment. Subsequently, miRNAs were extracted from plasma samples collected from healthy subjects (Con), patients with psoriasis vulgaris (Pso), and patients after acitretin treatment (Pso-A), after which miRNA sequencing (miRNA-Seq) was performed.

**Figure 1.**
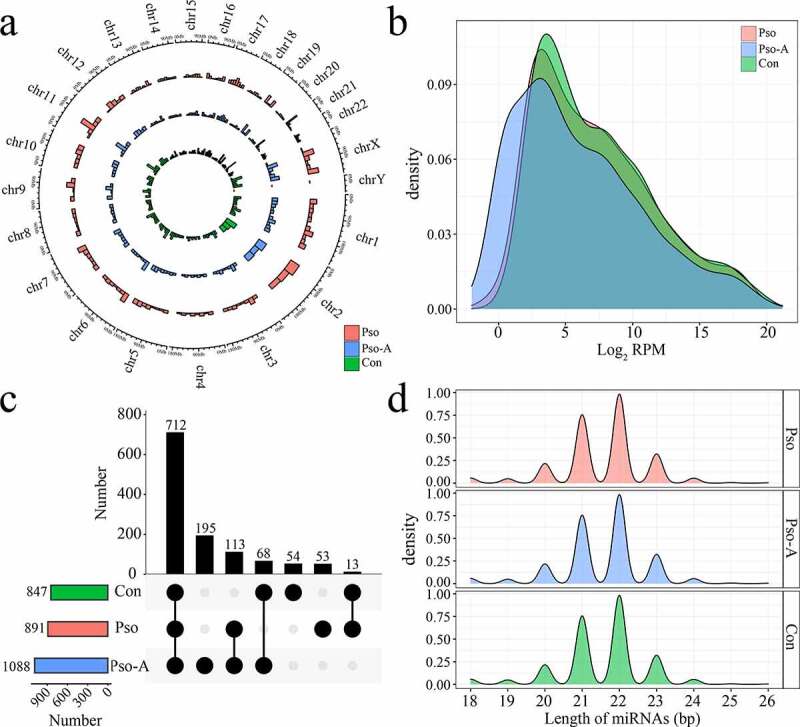
The basic characteristics of plasma-derived miRNAs from patients with psoriasis vulgaris (Pso) and patients treated with acitretin (Pso-A). a. The distribution of miRNAs, obtained by sequencing, on chromosomes. b. The distribution of miRNA expression. C. UpSet plot showed the common and unique miRNAs in the Con group, the Pso group, and the Pso-A group. Horizontal bars represent the total number of identified miRNAs within each group. Vertical bars represent the number of the common miRNAs in each group. The black original point below indicates the common situation of each group. (The vertical bars show the number of intersecting miRNAs between tissues, as denoted by the connected black circles underneath the histogram. The horizontal bars show the miRNA set size. D. The distribution of miRNA lengths

Through the miRNA-Seq data, we identified a total of 1683 miRNAs, of which 1209 were known miRNAs. Subsequently, the distribution, expression, and length of the identified and known miRNAs were statistically analyzed. With the exception of a small amount of miRNAs distributed on the Y chromosome, miRNAs of the three groups were distributed across all chromosomes (Figure 1(a)). The majority of miRNAs across the three groups demonstrated low expression levels (RPM<10) (Figure 1(b)), of which low expression miRNAs in the Con, Pso, and Pso-A groups accounted for 43.8%, 43.6%, and 42.0%, respectively, while high expression miRNAs (RPM>1000) accounted for 18.2%, 18.9%, and 18.7%, respectively. The length distribution of miRNAs was mainly concentrated at 21, 22, and 23 nt (Figure 1(d)). In addition, a total of 1 088 miRNAs were identified within the Pso-A group, 847 and 891 miRNAs were identified within the Con and Pso groups, respectively. In total, 712 miRNAs (58.9%) were identified across three groups (Figure 1(c)). There were 195 unique miRNAs within the Pso-A group, of which the majority were low expression miRNAs (94.9%).

### 3.2. Differentially expressed miRNAs analysis

**Figure 2.**
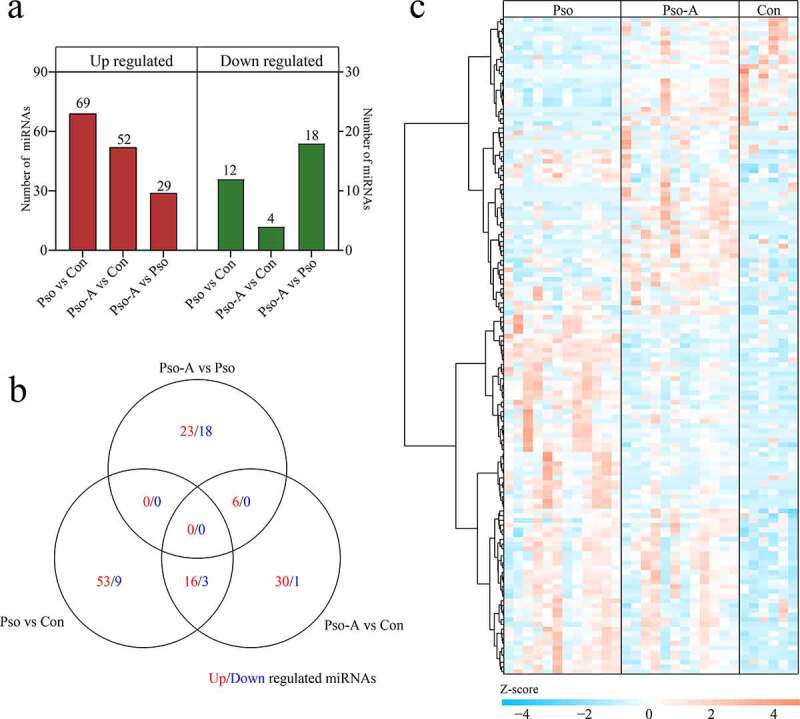
Differentially expressed miRNAs. a. The statistics of the number of differentially expressed miRNAs. b. Venn diagram demonstrates unique and common differential miRNAs between different groups. c. The clustering heat map of differential miRNA expression

To further investigate miRNAs change before and after acitretin treatment, based on the screening criteria of *P*< 0.05 and 2-fold difference, we assessed the levels of differentially expressed miRNAs between each group, and acquired 139 differentially expressed miRNAs (Table S3). Compared to the Con, the Pso obtained 81 differentially expressed miRNAs, of which 69 were up-regulated and 12 were down-regulated. Compared to the Con, the Pso-A acquired 56 differentially expressed miRNAs, of which 52 were up-regulated and 4 were down-regulated. Compared to the Pso, the Pso-A acquired 47 differentially expressed miRNAs, of which 29 were up-regulated and 18 were down-regulated (Figure 2(a)). The Venn diagram was used to analyze differentially expressed miRNAs among the comparisons. The results indicated that the number of differentially expressed miRNAs shared among the three groups was small (Figure 2(b)). A heat map was then used to comprehensively demonstrate expression of differentially expressed miRNAs. As shown in Figure 2(c), compared to the Con group, most miRNAs demonstrated an up-regulated expression within the Pso and Pso-A groups.

### 3.3. Circulating miRNA signature analysis

**Figure 3.**
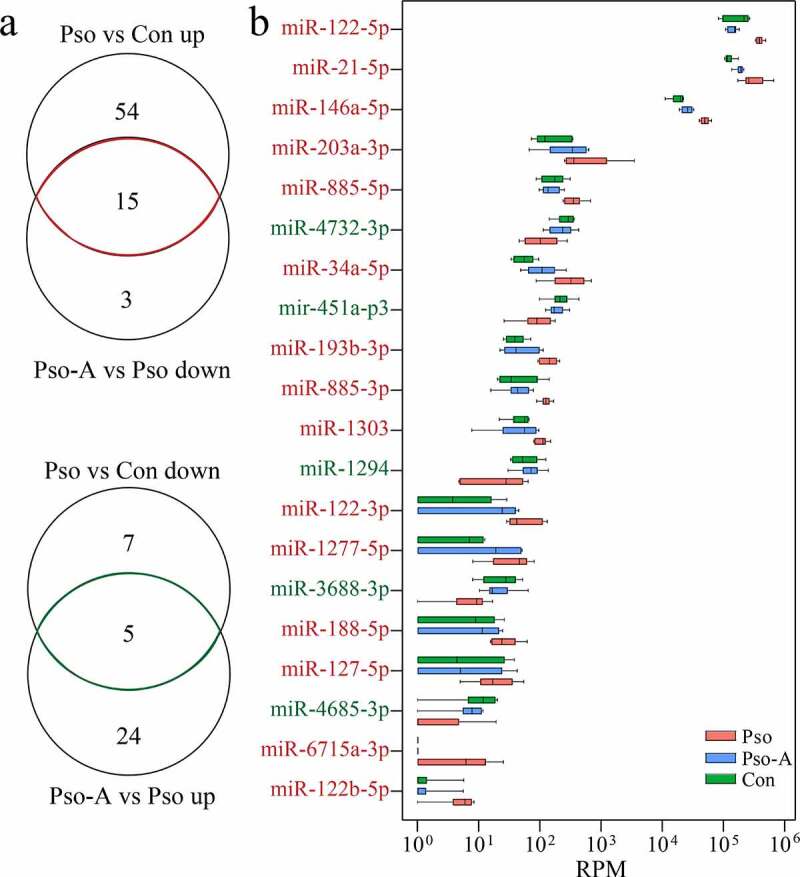
Plasma miRNAs are related to the efficacy of acitretin with regards to the treatment of psoriasis vulgaris. a. (Top) Venn diagram demonstrates the miRNAs that are up-regulated in the Pso group and down-regulated after acitretin treatment compared to the healthy group (Con). (Bottom) The miRNAs that are down-regulated in the Pso group and up-regulated after acitretin treatment compared with the healthy group. b. The 20 plasma-derived miRNAs that are related to psoriasis vulgaris after acitretin treatment in (a). The expression of miRNA is significantly different (Pso vs Con, Pso-A vs Pso; *P*< 0.05; |Log_2_ (fold change)| >1)

To determine the landmark miRNAs related to the therapeutic effect of acitretin, we further analyzed change characteristics of differentially expressed miRNAs among the three groups (Figure 3(a)). We identified 20 differentially expressed miRNAs, among which 15 were down-regulated and 5 were up-regulated within the Pso-A group (Figure 3(b)). Additionally, among the 20 miRNAs detected, three miRNAs were highly expressed, including miR-122-5p, miR-21-5p and miR-146a-5p.

### 3.4. Validation of miRNAs expression

**Figure 4.**
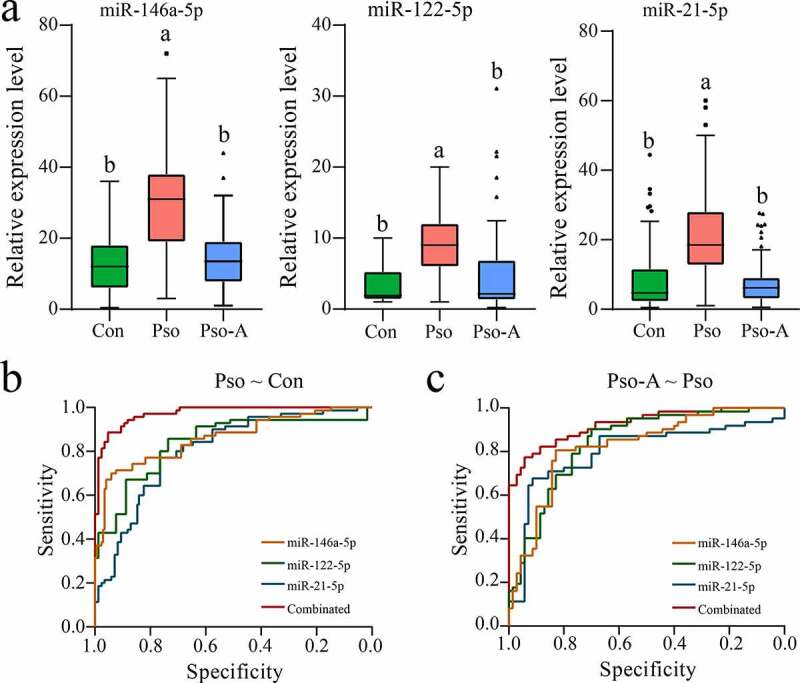
Validation analysis of the candidate miRNAs. a. The plasma-derived miRNAs from the Con (n = 80), Pso (n = 75), and Pso-A (n = 62) groups were analyzed using quantitative PCR. The significant difference between the two groups is evaluated through one-way ANOVA. The same letter represents insignificant difference, while different letters represent significant difference (p < 0.05). b. ROC analyzes the sensitivity and specificity of candidate miRNAs as diagnostic markers of psoriasis vulgaris. c. ROC analyzes the sensitivity and specificity of candidate miRNAs in order to evaluate the therapeutic effect of Acitretin

To further validate results of the miRNA screening, we collected a large number of plasma samples from patients with psoriasis vulgaris and patients treated with acitretin alone (the information of subjects was seen in Table S1). First, the expression levels of three miRNAs were verified using qRT-PCR. The results demonstrated that all three miRNAs were highly expressed in the Pso group, and their expression returned to low levels after acitretin treatment (Figure 4(a)), which is consistent with miRNA-Seq. Then, the ROC curve analysis further was performed to evaluate the accuracy and specificity of candidate miRNA as a treatment effect of acitretin. First, we evaluated whether miRNAs can be used as a disease diagnostic marker (Figure 4(b), Table 2). We discovered that the values of AUC of miR-146a-5p, miR-122-5p and miR-21-5p were 0.807 (95% CI: 0.738–0.875), 0.862 (95% CI: 0.802–0.921) and 0.840 (95% CI: 0.774–0.906), respectively, and that the combination of the three miRNAs had a higher AUC value (0.974). Similarly, higher sensitivity and specificity were obtained, at rates of 88.6% and 95.3%, respectively. Next, three miRNAs were utilized as indicators to determine the therapeutic effect of acitretin. The results demonstrated that the values of AUC of miR-146a-5p, miR-122-5p and miR-21-5p were 0.825 (95% CI: 0.753–0.897) and 0.831 (95% CI: 0.762–0.901) and 0.796 (95% CI: 0.713–0.879), respectively (Figure 4(c), Table 2). Similarly, combining the three miRNAs led to a higher AUC value (0.925), and the higher sensitivity (77.4%) and specificity (94.3%) were obtained. These results indicate that miR-146a-5p, miR-122-5p, and miR-21-5p have the potential to be utilized as markers for diagnosis of psoriasis vulgaris and evaluation of acitretin treatment effect.

**Table 2.** The areas under the curve (AUC), 95% confidence intervals (95% CI), sensitivity, and specificity of the receiver operating characteristic (ROC) curve analyses

Group	Parameter	miR-146a-5p	miR-122-5p	miR-21-5p	Combination
Pso ~ Con	AUC	0.807	0.862	0.84	0.974
	Std. Error	0.035	0.030	0.034	0.010
	*P* value	5.44E-11	1.04E-14	3.31E-13	3.69E-24
	95% CI	0.738–0.875	0.802–0.921	0.774–0.906	0.954–0.994
	Sensitivity (%)	71.4	85.7	77.1	88.6
	Specificity (%)	91.8	71.8	76.5	95.3
Pso-A ~ Con	AUC	0.825	0.831	0.796	0.925
	Std. Error	0.037	0.036	0.042	0.022
	*P* value	1.27E-10	5.42E-11	4.66E-09	3.87E-17
	95% CI	0.753–0.897	0.762–0.901	0.713–0.879	0.881–0.969
	Sensitivity (%)	80.6	90.3	66.1	77.4
	Specificity (%)	82.9	70.0	91.4	94.3

### 3.5. Acitretin may have a therapeutic function by down-regulating miRNA expression and inhibiting miRNA-mediated signaling pathways

**Figure 5.**
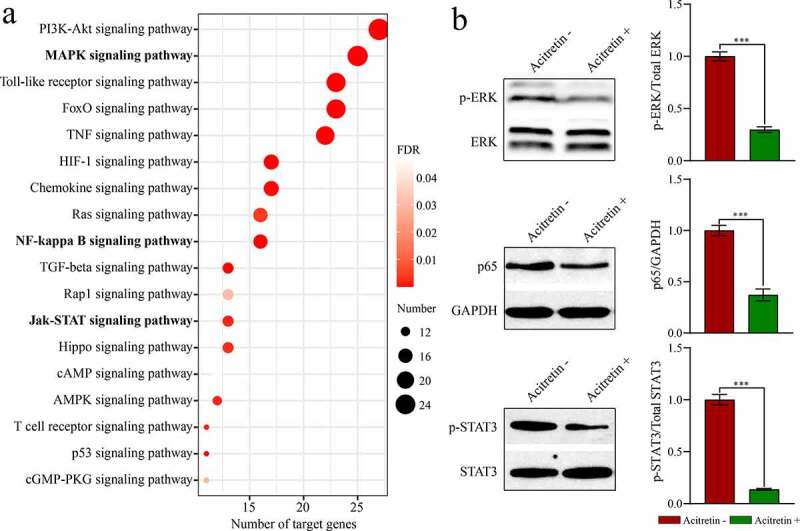
Acitretin has a therapeutic role by down-regulating the expression of miRNAs and inhibiting miRNA-mediated signaling pathways. a. KEGG analysis results of the target genes of the candidate miRNA. b. Western blot results validate the expression of p-STAT, p-ERK and p65 in HaCaT cells treated with acitretin *in vitro.*

To further evaluate the regulatory role of acitretin in the treatment of psoriasis vulgaris, we analyzed the target genes of the three miRNAs (miR-146a-5p, miR-122-5p, and miR-21-5p) and the possible signaling pathways mediated by miRNA. First, 73, 70 and 135 target genes of miR-146a-5p, miR-122-5p and miR-21-5p (Table S4) were acquired using the miRTarBase screening, respectively. Upon analysis of the signaling pathways involved in target genes through the DAVID database, 18 signaling pathways (Figure 5(a)) mediated by the miRNAs were acquired, such as the inflammation-related signaling pathways NF-kappa B and cell proliferation-related pathways MAPK and JAK-STAT.

To validate whether acitretin works by the signaling pathways, we used HeCaT cells to conduct *in vitro* experiments. We analyzed expression levels of the key proteins (ERK, STAT and p65) in the three signaling pathways (MAPK, JAK-STAT and NF-κB) using WB (Figure 5(b)). Compared to the control group, expression levels of p-ERK, p-STAT and p65 in the treatment group were down-regulated after treatment with acitretin for 24 h. These results suggest that acitretin can inhibit miRNA-mediated MAPK, JAK-STAT, and NF-κB signaling pathways by down-regulating miRNA expression, and therefore inhibiting proliferation and inflammatory response of keratinocytes.

## 4. Discussion

Herein, we analyzed the expression profile of plasma-derived miRNAs among patients with psoriasis vulgaris before and after treatment with acitretin using miRNA-seq. The expression levels of the three miRNAs (miR-146a-5p, miR-122-5p and miR-21-5p) were found to be significantly decreased after treatment with acitretin. Further analysis indicated that the three miRNAs can potentially be used as molecular markers to evaluate the therapeutic effect of acitretin. The analyses of signaling pathways mediated by miRNAs demonstrated that the three miRNAs were mainly involved in the MAPK, JAK-STAT, and NF-κB signaling pathways. Finally, we validated the three signaling pathways *in vitro*. We discovered that acitretin significantly inhibited the MAPK, JAK-STAT, and NF-κB signaling pathways by down-regulating miRNA expression.

MiR-146a is a marker of various inflammatory diseases, including rheumatoid arthritis and systemic lupus erythematosus [31,32]. The expression of miR-146a was increased in the skin and peripheral blood mononuclear cells (PBMCs) of patients with psoriasis, and miR-146a level was positively correlated to the severity of the disease [13,33]. Studies have reported that miR-146a can promote the occurrence of psoriasis by regulating macrophages [34], dendritic cells [35], Th1 cells [36], Treg cells [37] and Th17 cells [38]. Interleukin-17 (IL-17), an important pro-inflammatory cytokine, plays a key role in the occurrence of psoriasis [39,40]. Xia *et al*. demonstrated that in the skin and PBMCs of patients with psoriasis, high expression of miR-146a is positively correlated with IL-17 levels in the skin and serum, respectively [33]. Therefore, miR-146a may serve as a novel marker of psoriasis. Results from our study demonstrated that acitretin can decrease miR-146a-5p expression, suggesting that the therapeutic effect of acitretin on psoriasis may be related to miR-146a-5p.

MiR-21 is highly expressed in the T cells of patients with psoriasis [41]. However, miR-21 levels were decreased after treatment with ultraviolet B [42], suggesting that low expression of miR-21 is helpful in the treatment of psoriasis [43]. TNF-α can help promote the development of psoriasis, and the therapeutic effect of TNF-α inhibitors on psoriasis has confirmed this conclusion [44,45]. Blocking miR-21 using anti-miR-21 oligos or blocking TNF-α using the anti-TNF-α antibody etanercept can lead to the thickness decrease of psoriasis epidermal cells to varying degrees [46]. Previous studies have demonstrated that expression of miR-21 is significantly increased in 29 of the 32 psoriasis cases, and that expression of miR-21 is correlated to the low expression of metalloproteinase 3 (TIMP-3) [47]. Meanwhile, previous studies have demonstrated that miR-21-5p, an important regulatory factor in epidermal inflammation, can inhibit TIMP-3 [48]. The inhibited TIMP-3 can increase levels of TNF-α-converting enzyme (TACE) [49]. TACE, in turn, can promote the occurrence of psoriasis by releasing mediators of psoriasis, including TNF-α and EGF receptor (EGFR) [47]. Results from our study found that acitretin decreased levels of miR-21-5p, suggesting that this drug may inhibit the development of psoriasis vulgaris by regulating miR-21-5p levels.

Recent studies have discovered that miR-122-5p can have an effect on the occurrence of psoriasis [50]. The characteristics of psoriasis include excessive proliferation and differentiation of keratinocytes [51]. IL-22 is mainly produced by the activated Th1, Th17, and Th22, and can influence the keratinocytes [52]. MiR-122-5p has been confirmed to be involved in the keratinocyte response to IL-22 stimulation [50]. Sprouty2 (Spry2) belongs to the highly conserved Sprouty signal transduction family of proteins. The unique carboxyl-terminal cysteine-rich domain of Sprouty2 has an important role in inhibiting receptor tyrosine kinase (RTK) signaling pathway [53]. Additionally, Spry2 can the inhibit Erk/MAPK signaling pathway [54]. The Erk/MAPK signaling pathway has an important role in the pathogenesis of psoriasis [55,56]. It is demonstrated that IL-22 can increase expression of miR-122-5p, inhibit expression of Spry2, promote proliferation of keratinocytes, and eventually cause occurrence of psoriasis [50]. This study found that acitretin can significantly decrease expression of miR-122-5p and down-regulate MAPK signaling, indicating that acitretin can inhibit the occurrence of psoriasis vulgaris by regulating the MAPK signaling pathway mediated by miR-122-5p.

## 5. Conclusion

Collectively, in the treatment of psoriasis vulgaris, acitretin can exert therapeutic effects via inhibiting miRNA-mediated MAPK, JAK-STAT and NF-κB signaling pathways, thereby inhibiting the proliferation of keratinocytes and inflammatory response. Furthermore, landmark miRNAs can be used as potential molecular markers to evaluate the therapeutic effect of acitretin.

## Supplementary Material

Supplemental Material

## Highlights

- Three miRNAs were significantly decreased after acitretin treatment
- Targets of the miRNAs involved in MAPK, JAK-STAT, and NF-κB signaling pathways
- Acitretin can suppress miRNA-mediated signaling pathways by decreasing miRNAs
- Three miRNAs can be utilized to evaluate the therapeutic effect of acitretin
