# Supplementary material for: Circulating microRNA profile unveils mechanisms of action of acitretin for psoriasis vulgaris
Source: Bioengineered. 2021 May 11;12(1):1838–50. doi: 10.1080/21655979.2021.1925205 (PMC8806620; doi:10.1080/21655979.2021.1925205)
Supplement: Supplemental Material [file KBIE_A_1925205_SM2556.zip › Table S1.doc]

**Table S1. Clinical information from subjects for RT-PCR verification.**

| Parameters | Con Group | Pso Group | Pso-A Group |
| --- | --- | --- | --- |
|  | N=85 | N=70 | N=62 |
| Age, years, mean ± SD | 39.06 ± 15.02 | 41.03 ± 13.68*^ns/-^* | 41.17 ± 10.03*^ns/ns^* |
| Male, n (%) | 39 (45.88%) | 31 (44.29%)*^ns/-^* | 29 (46.77%)*^ns/ns^* |
| BMI, kg/m²,mean ± SD | 24.68 ± 4.38 | 23.00 ± 5.27*^ns/-^* | 24.07 ± 4.16*^ns/ns^* |
| Disease duration, years, mean ± SD |  | 13.33 ± 6.31 | 16.17 ± 7.03*^-/ns^* |
| Medication time, years, mean ± SD |  |  | 6.33 ± 4.46 |
| PASI |  | 18.25 ± 1.71 | 3.40 ± 1.93*^-/***^* |

*a/a*：statistically significant vs con group/statistically significant vs pso group, *ns*: not significant, *** *p* ≤ 0.001.
