# Supplementary material for: Circulating microRNA profile unveils mechanisms of action of acitretin for psoriasis vulgaris
Source: Bioengineered. 2021 May 11;12(1):1838–50. doi: 10.1080/21655979.2021.1925205 (PMC8806620; doi:10.1080/21655979.2021.1925205)
Supplement: Supplemental Material [file KBIE_A_1925205_SM2556.zip › Table S2.doc]

**Table S2. miRNA and internal reference sequences for RT-PCR experiments.**

| miRNA | Primer name | Sequence |
| --- | --- | --- |
| *miR-122-5p* | Forward | AGGCTGGAGTGTGACAATG |
|  | Reverse | GAGCAGGGTCCGAGGT |
|  | RT primer | GTCGTATGCAGAGCAGGGTCCGAGGTATTCGCACTGCATACGACCAAACA |
| *miR-146a-5p* | Forward | CGGCGGCTGAGAACTGAAT |
|  | Reverse | CAGTGCAGGGTCCGAGGTAT |
|  | RT primer | GTCGTATCCAGTGCAGGGTCCGAGGTATTCGCACTGGATACGACAAC |
| *miR-21-5p* | Forward | CAGCCACAAAAGAGCACAAT |
|  | Reverse | GGGGGTAGCTTATCAGACTGA |
|  | RT primer | CCTGTTGTCTCCAGCCACAAAAGAGCACAATATTTCAGGAGACAACAGGTCAACA |
| *U6* | Forward | CAAATTCGTGAAGCGTTCCATA |
|  | Reverse | AGTGCAGGGTCCGAGGTATTC |
|  | RT primer | GTCGTATCCAGTGCAGGGTCCGAGGTATTCGCACTGGATACGACAAAAAT |
